# Saunders’ Question Compends

**Published:** 1901-07

**Authors:** 


SAUNDERS' QUESTION COMPENDS.—ESSENTIALS OF THE DISEASES OF CHILDREN. By William M. Powell, M. D. Third edition. Thoroughly revised by Alfred Hand, Jr., M. D., Dispensary Physician and Pathologist to the Children's Hospital, Philadelphia. Two hundred and fifty pages; 12 mo. Philadelphia and London: W. B. Saunders & Co. Price, $1.00 net.

In the preparation of this little book the author has given very careful attention to a discussion of sympatomatology and therapeutics. However, he has not neglected the important questions of diet, general hygiene and nursing.

Dr. Hand in his review of the text has made some important alterations, and has added a number of new chapters, one of which deals with the artificial feeding of infants and fully covers the subject. W. B. R.

